# Leg ulcers in older people: a national study addressing variation in diagnosis, pain and sleep disturbance

**DOI:** 10.1186/s12877-016-0198-1

**Published:** 2016-01-21

**Authors:** Amanda Hellström, Camilla Nilsson, Annina Nilsson, Cecilia Fagerström

**Affiliations:** Department of Health and Care Sciences, Linnaeus University, Stagneliusg. 14, SE-392 34 Kalmar, Sweden; County Council of Värmland, Arvika, Sweden; County Council of Blekinge, Karlskrona, Sweden; Blekinge Institute of Technology, Blekinge Centre of Competence, Karlskrona, Sweden

**Keywords:** Leg ulcers, Older people, Pain, Sleep disturbances

## Abstract

**Background:**

Leg ulcers commonly emerge as a symptom of other comorbidities, often in older people. As a consequence of the ulcer, pain and sleep disturbances might occur. Due to the complex illness, the responsibility of treatment is unclear between health caregivers. The interaction between ulcer type, sleep and pain has not previously been investigated. This study aimed to explore pain in older men and women (65 years and older) with different diagnoses of leg ulcers and to investigate the associations of sleep disturbances and pain in people with leg ulcer diagnosis.

**Methods:**

The study used a cross-sectional design and data from the Swedish Registry of Ulcer Treatment, collected between May 2009 and December 2013. One thousand and eight hundred and twenty four people were included, and 62.9 % were women. The mean age was 83.4 years (SD 8.8). For the analyses, the chi-square test, Mann-Whitney U-test, t-test, one-way ANOVA and logistic regression was performed. Pain was measured by the Numeric Rating Scale (NRS), and sleep disturbances was assessed dichotomously.

**Results:**

We found the prevalence of pain intensity ≥ 5 on the NRS to be 34.8 % in those reporting pain. Additionally, the pain intensity was associated with the number of ulcers (*p* = 0.003). Sleep disturbances were associated with pain (*p* < 0.001) and were found in 34.8 % of the total sample. Although more women than men reported pain and scored higher on the NRS, no significant gender difference in sleep disturbances was found (*p* = 0.606). The mean NRS scores did not differ significantly between the ulcer types; however, arterial and venous-arterial ulcers increased the risk of sleep disturbances, as did higher pain scores.

**Conclusions:**

The majority of the participants were of advanced age (>80 years) and frequently suffered from pain and sleep disturbances. Further research is needed regarding pain, sleep and wound healing in the oldest old with leg ulcers. Ulcer pain sometimes appears to receive less attention in ulcer management, as do sleep disturbances, implying that individual needs might not be satisfactorily met. National guidelines in managing leg ulcers, which also consider consequences such as sleep disturbances, pain and discomfort, are needed.

## Background

Hard-to heal ulcers is a global health care problem, affecting 0.3 to 6 % of older people, resulting in economic consequences for society [1] as well as patient suffering [2]. Persons of advanced age are particularly at risk because of age-related diseases such as cardiovascular diseases as well as frailty [3]. Leg ulcer patients commonly report sleep disturbances, pain and itching [4, 5], which are symptoms related to each other frequently found in older patients. The types of leg ulcers are venous, arterial, venous-arterial and diabetes related; venous leg ulcers are the most normally observed type. There is frequently no aetiological diagnosis of a leg ulcer [6, 7], which complicates ulcer management and the treatment of symptoms associated with a specific ulcer diagnosis. Another complicating factor is that leg ulcers commonly emerge as a symptom from other comorbidities. These patients are consequently found throughout the health care system. As up till today, Sweden lacks a national strategy for the management of leg ulcers; and the care, diagnosing, treatment and follow up of those patients therefore depend on the competencies and skills at a local level, creating inequalities in care [8].

Pain sensation varies depending on the type of leg ulcer [4, 9]; however, studies comparing various leg ulcer diagnoses and the relationship of the diagnosis to pain and sleep disturbances are lacking. The prevalence of sleep disturbances [10, 11] and leg ulcers [1, 12] affect a significant number of older people and are more common among women. A gender perspective is regarded in this study investigating the occurrence of pain and sleep disturbance in relationship to a leg ulcer diagnosis.

Sleep disturbances are far more common in those with leg ulcers than in the general population, although the study finding this result was relatively small (*n* = 52) [5]. Satisfying sleep is important for the quality of life [13], as well as for recovery and wound healing [14]. Pain is a major concern for persons with leg ulcers, and it frequently leads to sleep deprivation [15]. Older people complain of pain more frequently than young people. However, chronic pain appear to be managed better by older people than by younger people because the sensitivity of pain is lower and the threshold of pain is higher in older people, which is hypothesized to depend on reduced tissue thickness and microcirculation changes in old age [16]. Herber et al. [17] found in their literature review that men complain of pain more than do women. Ulcer-related pain could be increased by anxiety, creating a cyclic pain process, leading to anxiety represented by intensification of pain [4]. Typically, sensory information is filtered during sleep; however, painful sensations indicating a threat of body homeostasis could trigger a rapid return to wakefulness [16], causing sleep disturbances. Pain and anxiety might negatively affect the sleep.

Taverner et al. [18] describe a trajectory of the pain associated with leg ulcers. At first, pain is predominantly of an acute nociceptive character; however, if an ulcer does not heal, persistent pain, with nociceptive and neuropathic properties, could develop. If the painful condition is not addressed at this stage, refractory chronic pain develops, which might cause insomnia, depression and suicidal ideation.[18]. Ebbeskog et al. [9] found differences in perceived ulcer pain, depending on the diagnosis, in a sample of 294 persons, of whom 92 % were >65 years of age. Pain in connection with arterial ulcers was more common and more intense. A later study [19] failed to confirm this difference; however, the study included only 96 persons from the general population. Paul [4] found venous ulcers to be the most painful. A study of 318 persons with venous ulcers [20] showed that ulcers were linked to several symptoms. The most common symptom was sleep disturbance, reported by 80 % of the sample, followed by pain and swelling/oedema.

Smokers or people with low physical activity have an increased risk of developing hard-to-heal ulcers [8]. Sleep is also found to be affected by nicotine such as an increase of difficulties initiating sleep, non-restorative sleep and difficulties waking up [21]. In a study by Tang and Sanborn [22] it was found that good sleep quality predicted higher levels physical activity the next day in persons with persistent pain lasting ≥6 months. Thus the interaction between sleep, pain and predisposing factors of leg ulcers is complex.

Previous research comprises of small studies [5, 19], studies that should be replicated for updating [9] or studies that did not investigate the associations between the ulcer diagnosis, pain and sleep disturbances in older people [4]. Such investigations are prompted because these conditions and symptoms could be expected to be most frequent in old age, and the proportion of older people in society is increasing. The aim of this study was to explore pain in older men and women (65 years or older) with various leg ulcer diagnoses. A further aim was to investigate the association of pain and disturbed sleep in leg ulcer patients.

## Method

The study used a cross sectional design and was based on data collected in the Swedish Registry of Ulcer Treatment (RUT). RUT is a national register developed in order to provide a more complete picture of ulcers and overcome organisational and professional boarders. The RUT was developed as a tool for the clinical assessment of hard-to-heal ulcers, treatment strategies and continuity of care, which was introduced nationally in Sweden in 2009; additional data are presented elsewhere [23, 24]. The 52 variables in the RUT include the patient’s history, ulcer history, pain, sleep, medical status, and ulcer treatment. The RUT was initiated because patients with hard-to-heal ulcers are frequently treated ineffectively and without a proper ulcer diagnosis [6, 7, 25]. This is a low priority patient group, in need of resource demanding care, often with an unclear responsibility of treatment between health caregivers [8, 24].

### Sample

The sample included 1824 persons 65 years or older registered in the RUT from the beginning of the study period (May 2009 to December 2013). Participants with a hard-to-heal leg or foot ulcer or a pressure ulcer are registered in the RUT by a nurse or a physician at two occasions: a first meeting in the primary care unit; and at a second time to evaluate the wound healing process and treatment [24]. The RUT includes data from the following types of Swedish care units: primary care units (approximately 75 %), dermatology departments, and community units.

### Data collection

Of the mandatory variables in the RUT, we selected the following variables from the registration at the first meeting: age, sex, smoking habits, physical function, ulcer pain and pain intensity, type of leg ulcer (diagnosis) and sleep disturbance. A leg ulcer is defined as an ulcer beneath the knee and is considered chronic if it is not healed within six weeks [26]. The six week limit is merely a lower threshold for an ulcer to be considered as hard-to-heal. The wounds are not actually assessed within six weeks in the register, but at the first meeting and a second time when the ulcer is healed. The median time for an ulcer to heal (in those registered) is 12 weeks.

### Procedure

Data for the present study was collected at the first meeting only, when diagnose and treatment strategy is decided. Nurses asked the questions and completed the registration forms. After the first meeting with the nurse, participants are assigned to one of the following treatments; local treatment of ulcer, local treatment of skin next to ulcer, larva, negative pressure or compression. No data on the treatment allocation of the participants were collected for this study.

The controlling factors regarding mobility were assessed with questions on the ability to walk, as follows: independent (able to walk without support); partly dependent (able to walk with support); and dependent (in need of a wheel chair). The questions regarding smoking habits determined whether the patient smoked, had never smoked or smoked previously. Age was divided into three groups (60–69, 70–79 and 80 years or older) in accordance with the classification by the WHO.

Pain was assessed by the following questions: whether the participants experienced pain (yes/no), and if so, the intensity of the pain using the 11-point Numerical Rating Scale (NRS), where 0 signifies no pain and 10 equals the worst imaginable pain [27, 28]. In cases in which the participant answered yes on the question regarding pain, the nurses were instructed according to the RUT registration form, to questioned he/she regarding sleep disturbances (“*Do you have sleep disturbances, yes/no”*).

The original ten types of ulcers registered in the RUT were reduced into the following categories of leg ulcers: arterial, venous, venous-arterial and other types. In the last category, pressure wounds, diabetic wounds, hydrostatic wounds, inflammation wounds, small vessel disease wounds, tumours of the skin and other types of ulcers were included.

### Data analysis

The statistical analyses were performed with IBM SPSS Statistics for Windows, Version 22.0. Armonk, NY: IBM Corp., USA. The descriptive statistics of the demographic variables are presented as quantities and percentages. The data on the nominal and ordinal level were analysed using the Pearson’s chi square test. The group comparisons of pain intensity were conducted by a t-test and one-way ANOVA. The logistic regression sub analysis further explored the effect of pain and a leg ulcer diagnosis on sleep disturbances. The response alternatives with the lowest association with sleep disturbances as well as men and the youngest subjects were used as references. The Hosmer and Lemeshow goodness-of-fit test was used to estimate the model extension fitting the sample [29]. The model was adjusted for gender and age. The mean pain intensity values are shown for the sample as a whole as well as for only those who had pain because pain is of particular clinical relevance. The level of significance was set at <0.05.

### Ethics approval

Participation in the RUT is voluntary, and the participants provided informed consent before any data were recorded [24]. The data included here were anonymized before delivery from the RUT. The authors have not been involved in the data collection process or the patient contact. No ethical approval was required due to local guidelines. In studies involving register data, the holder of the register has the right to decide the demands for gaining access to data. While some registers demand ethical approval, the RUT accepts a recommendation from the supervisor of the study.

## Results

Women represented the majority (62.9 %) of the registered persons (*n* = 1824). The ages ranged from 66 to 104 years and 65.4 % of the sample were 80 years old or older. There was a significant difference in the mean age between men and women (*p* < 0.001). Approximately 10 % of the sample smoked at the time of the study, and more men than women stated that they had previously smoked. The percentage of participants able to move without assistance was 46.7 % (Table 1).

**Table 1:** Description of the sample (*n* = 1824) according to gender in percentages; the number of individuals is presented within the brackets

	Men (*n* = 675)	Women (*n* = 1149)	Total (*n* = 1824)	*P*-value
Age				<0.001
60–69	10.1 (68)	5.1 (58)	6.9 (126)	
70–79	35.2 (238)	23.3 (268)	27.7 (506)	
≥ 80	54.7 (370)	71.6 (822)	65.4 (1192)	
Mean age (SD)	81.1 (8.8)	84.8 (8.6)	83.4 (8.8)	<0.001^a^
Smoker				<0.001
Current	10.5 (71)	9.2 (106)	9.7 (177)	
Never	51.0 (345)	69.5 (798)	62.7 (1143)	
Previous	38.5 (260)	21.3 (244)	27.6 (504)	
Mobility				<0.001
Without support	56.1 (379)	41.3 (474)	46.7 (852)	
With support	33.1 (224)	47.5 (546)	42.2 (770)	
Wheel chair	10.8 (73)	11.2 (129)	11.1 (202)	
Pain intensity (NRS)				0.011
0	53.0 (358)	46.0 (528)	48.5 (886)	
1	1.9 (13)	1.0 (12)	1.4 (25)	
2	3.1 (21)	4.0 (46)	3.7 (67)	
3	4.9 (33)	6.0 (69)	5.6 (102)	
4	6.2 (42)	5.9 (68)	6.0 (110)	
5	10.1 (68)	11.3 (130)	10.9 (198)	
6	3.8 (26)	4.9 (56)	4.5 (82)	
7	6.4 (43)	7.6 (87)	7.1 (130)	
8	6.4 (43)	6.4 (73)	6.4 (116)	
9	1.5 (10)	2.4 (27)	2.0 (37)	
10	2.8 (19)	4.5 (52)	3.9 (71)	
Mean score	2.58 (3.2)	3.07 (3.3)	2.89 (3.3)	0.002^a^
Sleep disturbances^b^				0.606
Yes	57.7 (213)	59.4 (422)	58.8 (635)	
No	42.3 (156)	40.6 (289)	41.2 (445)	
Leg ulcer diagnosis				0.002
Venous	37.4 (252)	45.1 (511)	42.2 (763)	
Arterial	11.4 (77)	9.4 (107)	10.2 (184)	
Venous-arterial	7.9 (53)	9.6 (109)	9.0 (162)	
Other types	43.2 (291)	35.9 (407)	38.6 (698)	
Number of wounds				0.673
One	90.2 (610)	90.9 (1045)	90.7 (1655)	
Bilateral	9.8 (66)	9.1 (104)	9.3 (170)	

Missing cases were found in three variables; diagnosis (*n* = 18) (0.9 %), age groups (*n* = 1) (0.05 %), pain intensity (*n* = 1) (0.05 %). Tests used: Chi-square

^a^ Independent samples t-test

^b^ Only persons answering the question regarding pain were able to answer the pain-related sleep disturbance question (*n* = 1080)

The most common leg ulcer diagnosis was that of a venous ulcer (42.2 %), where approximately every tenth participant had arterial (10.2 %) or venous-arterial ulcers (9.0 %). Other leg ulcer diagnoses represented 38.6 % of the total. One hundred and seventy participants (9.3 %) had bilateral ulcers, of whom 18 had a different diagnosis for each leg (Table 1). The diagnoses differed by gender in that more women had venous or venous-arterial ulcers, and men generally had arterial or other types of ulcers (*p*-value = 0.002).

One-half of the sample (51.5 %) experienced pain, most commonly at the level of 5 on the NRS. One thousand and eighty participants (59.2 %) answered the question regarding sleep disturbances, which means that more participants than those in pain were asked about their sleep, but not the whole sample. Of those asked 635 (58.8 %) reported sleep disturbances. No significant difference in the occurrence of sleep disturbances was found between women and men (*p* = 0.606). A gender difference was found in pain intensity (*p* = 0.011), for which women tended to score pain higher. According to the NRS (53/46 %), more men than women reported having no pain (Table 1).

In the sub analysis in which only those who reported pain (≥1 on the NRS) were included, the mean values of the pain intensity varied between diagnoses. Participants with multiple ulcers of different diagnoses scored highest (mean 7.58, SD1.24), followed by those with venous-arterial ulcers (mean 6.07, SD 2.25). The venous ulcer participants experienced pain (49.6 %), with a mean NRS score of 5.45 (Table 2). The participants with arterial and venous-arterial ulcers as well as bilateral ulcers reported more sleep disturbances than their counterparts (*p* < 0.001).

**Table 2:** Description of pain intensity in various ulcer diagnoses and the number of diagnoses

	Venous (*n* = 762)	Arterial (*n* = 184)	Venous-arterial (*n* = 162)	Other types (*n* = 698)	Lateral ulcer, One diagnosis (*n* = 1805)	Bilateral ulcers, different diagnoses (*n* = 18)
Pain (NRS) (%)						
0	50.4 (384)	27.2 (50)	38.9 (63)	54.9 (383)	48.8 (880)	29.4 (6)
1	1.3 (10)	1.6 (3)	0.0 (0)	1.7 (12)	1.4 (25)	0.0 (0)
2	2.9 (22)	4.3 (8)	3.1 (5)	4.6 (32)	3.7 (67)	0.0 (0)
3	6.6 (50)	6.0 (11)	6.2 (10)	4.4 (31)	5.7 (102)	0.0 (0)
4	7.3 (56)	6.5 (12)	4.9 (8)	4.9 (34)	6.1 (110)	0.0 (0)
5	9.8 (75)	16.8 (31)	13.0 (21)	10.0 (70)	10.9 (197)	5.9 (1)
6	3.9 (30)	6.5 (12)	8.6 (14)	3.6 (25)	4.5 (81)	5.9 (1)
7	7.6 (58)	8.7 (16)	8.0 (13)	5.7 (40)	7.0 (127)	17.6 (3)
8	5.6 (43)	9.2 (17)	8.6 (14)	5.4 (38)	6.1 (111)	23.5 (4)
9	1.8 (14)	3.3 (6)	1.9 (3)	1.6 (11)	1.9 (34)	17.6 (3)
10	2.6 (20)	9.8 (18)	6.8 (11)	3.2 (22)	3.9 (71)	0.0 (0)
Mean^a^ (SD)	5.45 (2.25)	6.04 (2.46)	6.07 (2.25)	5.42 (2.42)	5.59 (2.35)	7.58 (1.24)

The number of individuals are presented within the brackets (*n* = 1823, missing = 1)

^a^The mean score on the NRS was calculated for those rating pain as 1 or higher. There were no significant differences with regard to pain intensity between ulcer types when tested by one-way ANOVA with Tukey’s post hoc test. There was a significant difference in pain intensity between the number of ulcers when assessed by the t-test, *p* = 0.003

To explore the relationship between leg ulcers, pain intensity and sleep disturbances, the participants with or without sleep disturbances were compared. The chi-square test showed significant differences in the ulcer type and pain intensity between those with and without sleep disturbances, respectively. The number of ulcers was statistically significant for the pain intensity (*p* = 0.003, Table 2) and was not significant for sleep disturbances (Table 3).

**Table 3:** Associations of sleep disturbances, leg ulcer diagnosis, the number of ulcers and pain intensity in percentages

	Sleep disturbances	No sleep disturbances	*P*-value
Leg ulcer diagnosis^a^			<0.001
Venous	38.0 (237)	47.0 (208)	
Arterial	17.7 (110)	8.1 (36)	
Venous-arterial	13.2 (82)	7.2 (32)	
Other types	31.1 (194)	37.7 (167)	
Number of ulcers			0.510
One	88.7 (563)	90.1 (401)	
Bilateral	11.3 (72)	9.9 (44)	
Number of diagnoses			0.105^b^
One	98.3 (623)	99.6 (443)	
Two	1.7 (11)	0.4 (2)	
Pain (NRS)			<0.001
0	6.1 (39)	23.0 (102)	
1	0 (0)	5.6 (25)	
2	2.0 (13)	12.2 (54)	
3	5.8 (37)	14.6 (65)	
4	8.0 (51)	13.3 (59)	
5	19.1 (121)	17.3 (77)	
6	9.6 (61)	4.7 (21)	
7	16.9 (107)	5.2 (23)	
8	16.7 (106)	2.3 (10)	
9	5.4 (34)	0.7 (3)	
10	10.4 (66)	1.1 (5)	

The number of individuals is within the brackets

^a^ The participants with bilateral ulcers of different diagnoses were excluded, *n* = 18

^b^ Analysed by chi-square using a continuity correction

The association of disturbed sleep with pain intensity was explored with a logistic regression analysis having sleep disturbance as the dependent variable. The model was adjusted for a leg ulcer diagnosis, gender and age. Neither gender nor age had a significant influence on sleep disturbances, whereas a diagnosis of arterial (OR 2.41) and/or venous-arterial ulcer (OR 1.92) increased the odds for sleep disturbance. Pain intensity of 4 or higher was significantly associated with sleep disturbances, and the probability increased rapidly with the NRS score. The highest probability was found with a NRS score of 9 (OR 43.64, *p* < 0.001) (Table 4).

**Table 4:** Logistic regression analysis (*Enter*) of the factors associated with sleep disturbances, included in the analysis (*n* = 1066)

	Odds ratio	Confidence interval	*P*-value
Pain (NRS)			
1	0	0	0.998
2	0.17	0.30–1.27	0.190
3	1.52	0.87–2.65	0.145
4	2.37	1.39–4.06	0.002
5	4.11	2.55–6.63	<0.001
6	7.55	4.01–14.21	<0.001
7	12.80	7.06–23.20	<0.001
8	28.08	13.18–59.84	<0.001
9	43.64	9.90–192.29	<0.001
10	33.21	12.32–89.51	<0.001
Leg ulcer diagnosis			
Venous	0.96	0.69–1.34	0.795
Arterial	2.41	1.45–3.99	0.001
Venous-arterial	1.92	1.12–3.26	0.017
Age groups			
70–79 years	1.11	0.60–2.05	0.747
≥ 80 years	1.39	0.77–2.49	0.273
Sex			
Woman	0.96	0.70–1.31	0.782

References in the adjusted model: NRS 0, age 60–69 years, having other types of wounds and being male, with the Hosmer-Lemeshow goodness of fit of 0.947

## Discussion

This study reveals that, as a group, leg ulcer patients are of old age, and many of them have mobility problems, pain and sleep disturbances. Approximately 50 % of the sample experienced pain; 5 was the most common rating on the NRS for pain. Women reported pain more frequently and scored higher on the NRS. No significant differences in sleep disturbances were found between the genders, even if sleep disturbances was related to pain, particularly a high intensity of pain as well as to arterial and venous-arterial leg ulcers, which were more common in women.

Pain was more common in the participants with arterial ulcers and in those frequently reporting sleep disturbances, which is in accordance with a previous study by Ebbeskog et al. in older people [9]. In this study, pain was more common in specific diagnoses, and in spite of that the mean pain intensity varied by ulcer type; the difference in intensity between the four ulcer types was not significant. Instead, the number of ulcers was a vital matter for the experience of pain and sleep disturbances. Closs et al. [30] study (*n* = 52) only included venous and arterial ulcers; however, they showed that people with arterial ulcers rated their pain higher than those with venous ulcers. In addition they found that the pain characteristics varied between the two, with arterial ulcers being associated with a sharp and intense pain.

This study revealed a significant difference between men and women in pain intensity, were mean NRS score of the women was higher. This is in line with previous research comparing pain perception in men and women [31] Gender roles could possibly explain this finding because expressing pain is more acceptable in women than in men [32], which could imply that severe pain in men is underreported. Surprisingly there was no significant difference between genders regarding sleep disturbances. Previous research points at sleep disturbances are more common in women [33]. A possible explanation of our finding, needing further investigation, is that pain did not increase the number of women with sleep disturbances in a noticeable way, because a great number of the women might already be suffering from disturbed sleep for other reasons. In men, where the prevalence of sleep disturbances usually is lower than shown in our study, pain could be a decisive factor of the high prevalence, levelling out the number of men and women with sleep disturbances.

Higher pain intensity was associated with increasing odds of having sleep disturbances, and the odds increased rapidly with the NRS score (NRS ≥ 4). This finding is in concordance with results from a previous study [5], which showed that pain (rated on a five-grade scale) negatively affected sleep. Leg ulcers are frequently perceived to be more painful in the afternoon or evening and could thereby have an increased effect on sleep [18, 30, 34]. Of the patients with variously diagnosed bilateral ulcers, 70.5 % scored 5 or higher on the NRS. Among those with arterial ulcers, the corresponding figure was 54.3 %. Additionally, having arterial or venous-arterial ulcers affected sleep negatively in the bivariate and logistic regression analyses; the occurrence of bilateral ulcers did not have the same effect. The study included a limited number of individuals with variously diagnosed bilateral ulcers (*n* = 18), which resulted in a large confidence interval. This result should be interpreted with caution. The various types of ulcers affect sleep differently, and the sleep status of patients with arterial or venous-arterial ulcers should receive attention. Taverner et al. [18] showed that people with leg ulcers sometimes hesitate to visit care professionals until they experience persistent pain. By then, a chronic pain condition could have been established, with additional consequences that affect health and sleep [18]. Also, previous research show that pain often is unrecognized and is not assessed consequently in leg ulcer patients [8]. It is of most importance that these patients are assessed for pain and sleep disturbances soon after a leg ulcer diagnosis, and that pain and sleep is assessed at follow-ups.

Of those participants answering the question regarding sleep, 58.8 % claimed to have sleep disturbances. A higher prevalence of sleep disturbance among elderly people with pain has been described previously [35], although it was reported by 39.9 % among patients with pain, which is somewhat lower than was found in our findings. Upton and Andrews [5] found 69 % of people aged ≥50 with leg ulcers had poor sleep; their study was quite small (*n* = 52). Foley et al. [36] showed that among a community sample of 1506 persons aged 55–84 years nearly 40 % of those with major comorbidity compared to 10 % of those without medical conditions perceived impaired sleep quality. Most common were sleep disturbances in connection with bodily pain, heart disease, depression or cognitive impairments [36]. In line with these studies, our study reveals a higher prevalence of disturbed sleep in people with pain than among older people in general. The prevalence of sleep disturbances among older people without persistent pain varies between 21.7–35 %, depending on the measure that was used [37, 38]. Sleep disturbances could lead to an impaired healing process, causing prolonged suffering. Altemus et al. [39] suggested that short-term loss of sleep might affect the skin barrier function, in young women, and sleep-deprived rats are found to have impaired healing [14]. Studies investigating the effect of sleep loss on defence systems are ambiguous because it is unclear whether sleep loss or the stressful methods used to induce sleep loss causes the resulting negative effects. However, loss of sleep could not be ruled out as a possible cause of alterations in body defences [40].

### Strengths and limitations

The study has several strengths, including the sample. The prevalence of ulcer wounds is low and is found in 0.6–3 % of those aged over 60 years [41]. Because the RUT is based on a country wide national register, a large sample size and a variety of diagnoses was possible in the study, which is in contrast with most leg ulcer studies, which focus solely on one diagnosis in a limited number of patients. The large sample size allowed for comparison and provided deeper knowledge of the associations between a leg ulcer diagnosis, pain and sleep disturbances in men and women. All the health care personnel working on the RUT are trained and register similar findings according to a pre-defined protocol.

This study includes also some limitations. In the RUT one binary question was used to measure sleep disturbances. This design provides information regarding the presence of sleep disturbances, not their character, duration, frequency or severity. Sleep disturbances consist of a wide variety of symptoms and the perception of disturbing elements might vary among individuals [42, 43]; the use of one question affects the precision of the measure. Further exploration of the types of sleep disturbances the participants experience would have been optimal. The study uses the question regarding sleep as a supplementary question to the investigation of the presence of pain. Sleep disturbances could exist without concurrent pain. Pain is a common cause of sleep disturbances; however, Andrews and Upton [5] found that people with ulcers reported sleep disturbances because of nocturia or anxiety. Additionally, the pain questions showed some inconsistencies. A few participants recording ‘no pain’ were questioned regarding their sleep (*n* = 18). Of those 18 people, seven (38.9 %) reported sleep disturbances. Of those rating their pain intensity as 0 (no pain), 39 (4.4 %) reported sleep disturbances, which indicates that sleep disturbances could exist in the sample cohort regardless of pain, as noted by Upton and Andrews [5]. Considering the total sample of our study; solely 59.2 % were asked about their sleep. The prevalence of sleep disturbances in leg ulcer patients, regardless of pain, is therefore still unknown. Of the participants reporting no pain, 15 scored higher than 0 on the NRS, and of those, ten scored 5 or higher on the NRS, which might indicate a misunderstanding of the questions and an underestimation of the number of participants experiencing pain in connection with an ulcer. These few participants, representing 1 % of the sample, are expected to have had a limited effect on the registered results.

The validity of using the NRS in older adults with chronic wounds, including hard-to-heal ulcers, could be questioned. The NRS was developed for acute pain; it has been validated as a measure of measuring chronic pain as well. Additionally, the NRS is more reliable than the Verbal Rating Scale (VRS), which is commonly used to assess pain intensity [27]. Because of the ambiguous answers regarding pain and its intensity, the validity of using the NRS in older adults could be insufficient. A study that tested the psychometric properties of the NRS [28] showed that it was suitable for assessing pain intensity among people 60 years of age or older. Failure to complete the rating increased with higher age, particularly in those over 80 years of age [28]. In this study, 65.4 % of the participants were ≥80 years of age, which could suggest that several participants had difficulties answering the questions. However, since nurses asked the questions in a patient interview and completed the registration forms, this should have minimized the risk of internal dropouts and misunderstandings during the registration. Another aspect regarding ulcer pain is that it may vary from day to day, during the course of the day or depending on the weather or season [15]. This implies that for a more precise estimation of the impact pain has on daily life, several measurements are required.

## Conclusion

This study shows that the majority of individuals with hard-to-heal leg ulcers are of advanced age (>80 years) and have impaired mobility. Individuals in this age group frequently suffer from health problems including pain and sleep disturbances, which could affect their overall well-being as well as the healing process. Further research should focus on the relations between pain, sleep and wound healing in the oldest old with leg ulcers. Also the impact of leg ulcers on quality of life and well-being needs more attention in research. Ulcer pain sometimes appears to receive less attention in ulcer management, and thus individual needs might not be satisfactorily met. A careful anamnesis of the ulcer type, number of ulcers, and presence of sleep disturbances is required for personalized care that considers the health needs of each individual. There is a need for national guidelines in managing leg ulcers, which also consider consequences such as sleep disturbances, pain and discomfort such as odour, swelling and oedema, which all are factors that may impact well-being in those patients.

## Availability of data/materials

The data used for this study was provided by the register RUT. We do not have the authority to share that data, since we are not the holders of the register. Interested researchers are urged to contact Rut M Öien at www.rut-europe.eu in order to access data.
